# Un AVC ischémique a IRM cérébrale normale: à propos d’un cas

**DOI:** 10.11604/pamj.2016.25.22.10413

**Published:** 2016-09-26

**Authors:** Glorien Lemahafaka, Ansoumane Camara, Lala Rajaonarison, Francis Vallet

**Affiliations:** 1 Service de Neurologie, Centre Hospitalier de Pontarlier, France; 2 Service de Neurologie, Centre Hospitalier Joseph Raseta Befelatanana, Madagascar

**Keywords:** AVC, ischémie cérébrale, IRM cérébrale, Stroke, brain ischemia, brain MRI

## Abstract

L'IRM cérébrale reste un examen de référence pour diagnostiquer un accident vasculaire cérébrale. La sensibilité au cours d'un accident vasculaire cérébrale à la phase aigüe n'atteint pas toujours 100%. Nous rapportons un cas d'un accident vasculaire cérébral ischémique à IRM cérébrale initiale normale. L'IRM cérébrale normale à la phase précoce d'un accident vasculaire cérébrale pose un doute sur l'indication de la thrombolyse qui est le traitement de référence en cas d'embole cérébrale. La répétition d'un examen avec coupe fine, en séquence de diffusion B2000 permettrait de mettre en évidence l'infarctus de très petit volume non visualisé par IRM cérébrale conventionnelle.

## Abstract (en)

MRI remains the current gold standard imaging modality to diagnose ischemic stroke. MRI sensitivity for acute ischemic stroke detection does not always reach 100%. We report the case of a patient with ischemic stroke despite normal initial brain MRI. Normal brain MRI during the early phase of ischemic stroke raises doubt about the indication for thrombolysis which is the treatment of choice for cerebral embolism. The repetition of fine cut examination by B2000 diffusion weighted imaging (DWI) enables the early detection of small volume infarction not visualized by conventional brain MRI.

## Introduction

Un accident vasculaire cérébral est défini par l'installation brutale d'un déficit neurologique focalisé d'origine vasculaire qui est ischémique (85%) ou hémorragique (15%) [1, 2]. L'ischémie cérébrale est la conséquence d'une réduction ou interruption brutale du débit sanguin cérébral qui est normalement supérieur à 50ml/100g de tissu cérébral/mn, souvent secondaire à une occlusion artérielle à destination cérébrale [2, 3]. L'IRM cérébrale reste la modalité de choix pour le diagnostic de l'ischémie cérébrale car elle visualise simultanément le foyer ischémique quel que soit sa taille ou sa topographie dans tous les axes artériels [1]. Cette hypothèse n'est pas toujours valable en pratique, nous rapportons un cas inhabituel d'ischémie cérébrale à IRM cérébrale normal à la phase aigüe dans le but de discuter le diagnostic par rapport à la revue de la littérature. Contexte: Il s'agit d'un cas clinique observé au service de neurologie de Pontarlier au mois de juillet 2016.

## Patient et observation

**Figure 1:**
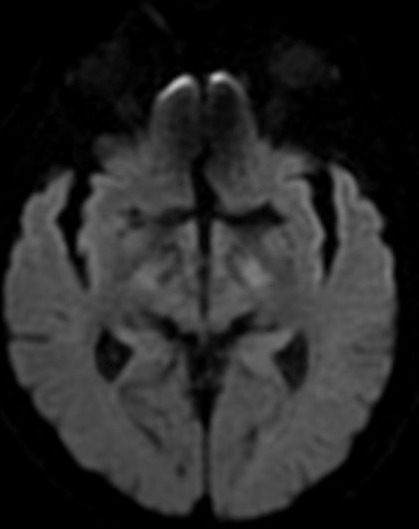
IRM cérébrale de diffusion initiale montre une absence de signe d’infarctus cérébrale

**Figure 2:**
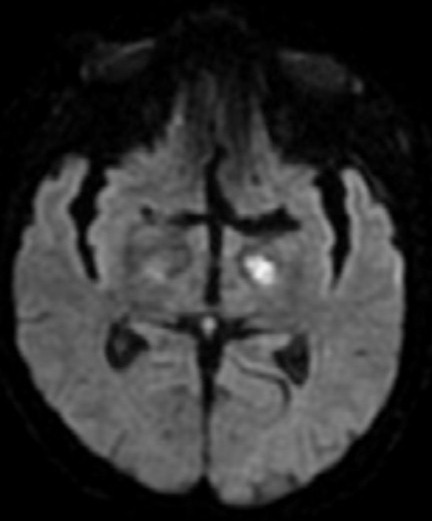
IRM cérébrale en séquence de diffusion montre in infarctus de la branche profonde de l’artère sylvien gauche

**Figure 3:**
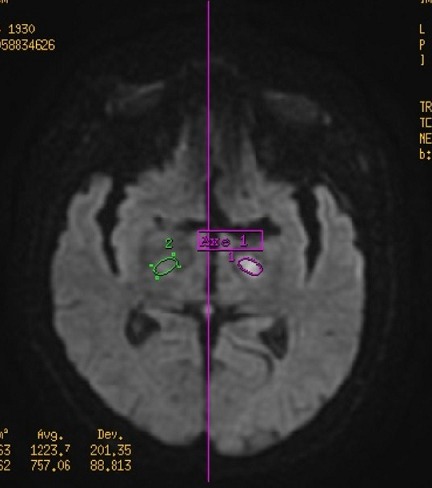
IRM cérébrale montre une baisse significative de coefficient de diffusion confirme le caractère infarcis du territoire de l’artère choronoidienne antérieure gauche

Un patient âgé de 86 ans, agriculteur retraité, ayant comme antécédents personnels : une hernie hiatale, deux épisodes d'ictus amnésiques. Il avait présenté soudainement une difficulté à s'exprimer par recherche des mots à prononcer ou manque de mot, un langage incompréhensible sans trouble de compréhension le 06.07.2016 à 9h du matin. Arrivé aux urgences à 9h40, les paramètres vitaux sont normaux, l'examen neurologique retrouve un patient conscient, score de Glasgow à 15/15, une aphasie non fluente, une jargonophasie, une anomie, une hyposensibilité de l'hémicorps droit. Le score NIH initial est évalué à 2. Le bilan biologique initial ne montre rien de particuliers sauf une hypercholestérolémie totale à 2,87g/l, HDL-cholestérol à 0,39g/l, et LDL-cholestérol à 1,26g/l. L'IRM cérébrale réalisée à 2h (11h00) du début des signes montre une absence d'anomalie en pondération de diffusion, le coefficient de diffusion indifférent. Les autres pondérations étant libres de tout processus évolutif. Etude vasculaire 3D TOFF sans particularité (Figure 1). Devant la persistance des symptômes, le deuxième IRM cérébrale de contrôle a été faite après 24h, objective un hypersignal thalamique gauche en pondération de diffusion et T2 FLAIR, avec baisse de l'ADC qui confirme un infarctus du territoire de l'artère choronoidienne antérieure gauche (Figure 2, Figure 3). L'échographie et doppler des vaisseaux du cou montrent des lésions athéromateuses des deux carotides internes non hémodynamiquement significative. Artère vertébrale droite hypoplasique. Le monitoring cardiaque montre une absence de trouble du rythme emboligène. L'échographie cardiaque transthoracique normale. Nous avons mis le patient sous protocole cérébrolésé selon la recommandation de HAS. Il a bénéficié d'un bolus initial de Kardégic à 300mg en IV avec relais antiagrégant à 160mg/j en per os, d'un Statine, monitoring et surveillance journalière de score NIH. L'évolution est favorable par régression complète des symptômes, le score NIH est à 0 le cinquième jour. Le patient a pu regagner son domicile, l'évaluation orthophonique ne retient pas dans la suite de la prise en charge.

## Discussion

Il s'agit d'une forme inhabituelle d'un infarctus cérébrale. L'IRM cérébrale réalisé 2h après le début des symptômes ne détectent aucune anomalie de parenchyme cérébral alors que les symptômes neurologiques typiques d'un accident vasculaire cérébral persistaient. Dans la littérature les différents cas rapportés, montre pourtant que l'IRM cérébrale pose le diagnostic d'ischémie cérébral dès la première minute, elle évalue la possibilité d'une thrombolyse, puis détermine le pronostic et participe au diagnostic étiologique [4, 5]. La sensibilité diagnostique de l'imagerie de diffusion en résonnance magnétique pour détecter une lésion ischémique cérébrale dans les 6 premières heures est d'environ 90%, ce qui veut dire que dans 10% des cas l'IRM cérébrale peuvent être normale [2, 4]. Les faux négatifs peuvent exister surtout en cas de lésion de volume très réduit d'environ 1 ml, vus très précocement. Donc l'IRM cérébrale peut être normal (ou subnormal), ce qui peut conduire à des erreurs diagnostiques [2, 3]. L'IRM en coupe fine avec séquence de diffusion B2000 améliore la sensibilité de l'IRM pour diagnostiquer un AVC ischémique en phase aigüe non diagnostiqué par l'IRM cérébrale conventionnelle.

## Conclusion

L'IRM cérébrale peut être normale au cours d'un infarctus cérébrale de volume très réduit, vue très précocement. La répétition de l'IRM cérébrale avec coupe fine en séquence B2000 permettrait de mettre en évidence les lésions ischémiques de plus petit volume alors que l'IRM cérébrale conventionnelle peut être négative.
